# The Obstetric Consequences of Female Genital Mutilation/Cutting: A Systematic Review and Meta-Analysis

**DOI:** 10.1155/2013/496564

**Published:** 2013-06-26

**Authors:** Rigmor C. Berg, Vigdis Underland

**Affiliations:** Norwegian Knowledge Center for the Health Services, P.O. Box 7004, St. Olavsplass, N-0130 Oslo, Norway

## Abstract

Various forms of female genital mutilation/cutting (FGM/C) have been performed for millennia and continue to be prevalent in parts of Africa. Although the health consequences following FGM/C have been broadly investigated, divergent study results have called into question whether FGM/C is associated with obstetric consequences. To clarify the present state of empirical research, we conducted a systematic review of the scientific literature and quantitative meta-analyses of the obstetric consequences of FGM/C. We included 44 primary studies, of which 28 were comparative, involving almost 3 million participants. The methodological study quality was generally low, but several studies reported the same outcome and were sufficiently similar to warrant pooling of effect sizes in meta-analyses. The meta-analyses results showed that prolonged labor, obstetric lacerations, instrumental delivery, obstetric hemorrhage, and difficult delivery are markedly associated with FGM/C, indicating that FGM/C is a factor in their occurrence and significantly increases the risk of delivery complications. There was no significant difference in risk with respect to cesarean section and episiotomy. These results can make up the background documentation for health promotion and health care decisions that inform work to reduce the prevalence of FGM/C and improve the quality of services related to the consequences of FGM/C.

## 1. Introduction

Various forms of female genital mutilation/cutting (FGM/C) have been performed for millennia [1] and continue to be prevalent in many parts of the world, especially in Africa [2]. The procedure, variously termed across disciplines and perspectives, is classified by the World Health Organization into four types depending on the extent of tissue removed, where type III, infibulation, is the most extensive [3]. The procedure of infibulation derives its name from the Roman word *fibula* (clasp), which was fastened through the prepuce of men and labia of women to enforce chastity. While a range of socioreligious issues foster the practice, to this day a conviction that FGM/C is necessary to control women's sexuality exists in many practicing communities [2, 4]. Studies have also revealed that many members of practicing communities believe that the procedure ensures safe labour [5, 6].

Survey data document that across the world, between 100 and 140 million girls/women are presently living with FGM/C [3] and its health consequences. The medical and related health consequences following FGM/C on a short- and long-term basis have been broadly investigated. Obermeyer's two reviews of the consequences of FGM/C for health and sexuality are informative, highlighting that there exist statistically higher risks for some but not all investigated types of health conditions [7, 8]. A more recent systematic review of the sexual consequences from FGM/C included meta-analysis results, showing that women with FGM/C were more likely than women without FGM/C to experience pain during intercourse, reduced sexual satisfaction, and reduced sexual desire [9]. The medical profession has been particularly concerned about the risk of adverse obstetric events for women who have undergone FGM/C. The WHO literature report of the health complications from FGM/C which highlighted sequela in childbirth [10] provides the most comprehensive summary of such complications. The review was not systematic, according to today's internationally recognized standards [11–13], since there were no explicit eligibility criteria, quality appraisal, or data synthesis. However, in the WHO report, it is concluded that “the serious obstetric consequences of FGM, when it is performed prior to the index pregnancy, are mainly due to the scarring resulting from FGM” [10, page 12]. In fact, a range of studies suggests that the most plausible pathway of effect between FGM/C and obstetric harm is inelastic scar tissue [14–20]. However, divergent results among such studies and statements by scholars, physicians, and policy experts claiming that “reproductive health and medical complications associated with female genital surgeries in Africa are infrequent events” [21, page 22] have called into question whether FGM/C is associated with obstetric consequences for women.

To address systematic review omissions in the literature, clarify the present state of empirical research, and enable the quantification of the obstetric health impacts of FGM/C at the population level using burden of harm and comparative risk assessment methodology, we conducted a systematic review of the scientific literature and quantitative meta-analyses. To the best of our knowledge, this is the first meta-analysis to summarize the evidence for associations between FGM/C and outcomes related to maternal obstetric health. This systematic review is an abridged and revised communication of a technical report conducted at the Norwegian Knowledge Centre for the Health Services [22].

## 2. Materials and Methods

We followed an open process for this systematic review with input from stakeholders and a protocol, published in PROSPERO, that followed standards for systematic reviews [11, 12, 23]. A full technical report with detailed search strategies, methods, and evidence tables is available elsewhere [22].

### 2.1. The Literature Search

We conducted comprehensive and systematic searches in MEDLINE (Appendix A), African Index Medicus, British Nursing Index and Archive, CINAHL, the Cochrane Library (Cochrane Central Register of Controlled Trials, Cochrane Database of Systematic Reviews, Database of Abstracts of Reviews of Effects, and Health Technology Assessment Database), EMBASE, PILOTS, POPLINE, PsycINFO, Social Services Abstracts, Sociological Abstracts, and WHOLIS for studies published in January 2012. To maximize the sensitivity of database searches, we neither applied methodology search filters nor restricted the searches to any specific languages or publication dates. We supplemented the electronic database searches with searching in sources for the grey literature (OpenGrey, OpenSigle, OAIster), browsing websites of international organizations that are engaged in projects regarding FGM/C, searching reference lists of relevant reviews and all included studies, and communicating with experts in the field.

### 2.2. Study Selection

The processes of study selection, methodological quality appraisal, and data extraction were conducted by two investigators, first independently and then jointly. Discrepancies were resolved through discussion and further inspection of the texts. If consensus had not been reached, we would have consulted a third person, but this was not necessary.

Two investigators first screened titles and abstracts. We retrieved the full text of potentially relevant studies, reviewing each article using a standardized form with a priori eligibility criteria. We included studies providing quantitative data on physical consequences if they were of any study design, except qualitative studies. Study design features (as defined in the Cochrane glossary [11]), not study design labels, were used to designate the studies. Methodological study quality was not a basis for inclusion/exclusion. Eligible population was women who had been subjected to any type of FGM/C, and the exposure or event of interest was FGM/C, classified as type I to type IV according to the WHO modified typology [3]. We excluded consequences of a woman's FGM/C on other individuals, such as effects on babies during birth. Both studies with and without a comparison group were considered. Concerning outcomes, the range of physical outcomes were included. Given the volume of data deemed eligible (185 studies), in this communication, we report on obstetric consequences in women with FGM/C compared to women with no FGM/C, including the obstetric outcomes most frequently reported. Other outcomes and results will be detailed in forthcoming technical reports available from the Norwegian Knowledge Centre for the Health Services.

### 2.3. Methodological Quality Assessment and Data Extraction

Two investigators rated the methodological quality of included studies using design specific checklists and extracted data using a standardized form. We extracted information on study characteristics, sample, exposure to FGM/C, outcomes, and results. Outcomes were general and specific measures of consequences following FGM/C (e.g., episiotomy, lacerations). When outcome data were missing in the publication, we contacted the corresponding author(s) via e-mail and requested that they send us the data. We grouped the data according to outcomes across types of studies, prioritizing in this communication to detail results from studies with highest internal validity (studies which compared groups of women with FGM/C to women without FGM/C).

### 2.4. Data Analysis and Rating the Body of Evidence

We conducted meta-analyses in RevMan v5.2.4 [24] when studies were sufficiently similar in terms of design, population, exposure, and outcomes. We combined risk ratios for dichotomous outcomes using the Mantel-Haenszel random-effects model, which weighted studies by the inverse of their variances, giving more weight to precise studies. Continuous outcomes were combined using inverse-variance random effects meta-analysis, calculating mean differences with 95% CIs. We quantified statistical heterogeneity using the *χ* ^2^ and *I* ^2^ statistics where a high value shows that most of the variability across studies is due to heterogeneity rather than to chance. We conducted sensitivity analyses for study type and outcome (definition and measurement) when possible. For clarity of presentation, when such tests showed no significant differences we present the final meta-analysis result. We calculated absolute risk differences for the adverse events to enhance interpretation of results. It shows the additional absolute risk of obstetric harm when FGM/C had been carried out.

Lastly, two investigators independently evaluated strength of evidence using the Grading of Recommendations Assessment, Development and Evaluation approach (GRADE), with GRADE-Profiler v3.6 [25], to assess the extent to which we could have confidence in the effect estimates [26]. For each outcome eligible for meta-analysis, we examined five domains: methodological quality of study, consistency, directness, precision, and publication bias. If admissible, we would have examined also strength of evidence of association, evidence of a dose-response gradient, and all plausible confounders. In the GRADE system, randomized trials always begin with a “high” strength of evidence that can be downgraded, and observational studies begin with a “low” strength of evidence that can be further downgraded but can also be upgraded (see [27] and http://gradeworkinggroup.org/). In this systematic review, all included studies were necessarily observational; thus, the evaluation of evidence started from a position of low quality. We used the standard definitions in grading the quality of the evidence, assigning an overall grade of “high,” “moderate,” “low,” or “very low” strength of evidence [27].

## 3. Result and Discussion

**Figure 1:**
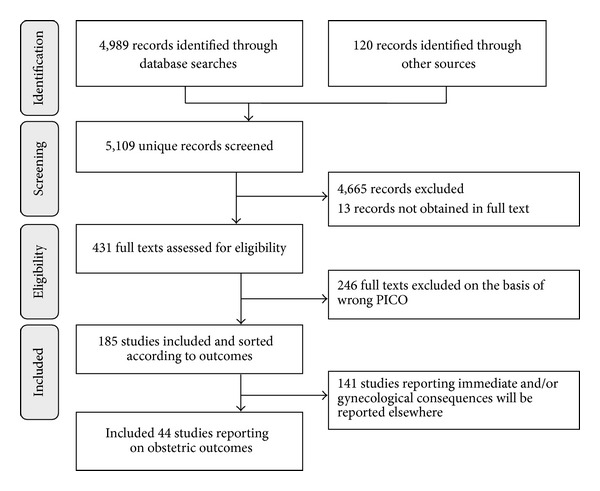
PRISMA flow diagram of the literature reviewing process.

A total of 5,109 unique study reports were identified (Figure 1). After sorting eligible studies according to outcomes, we included 44 primary publications reporting on obstetric outcomes: 21 comparative studies [14–20, 28–47], 7 single group cross-sectional studies [48–54], 5 case series [6, 55–58], and 4 case reports [59–62].

### 3.1. Description of the Included Literature

In line with the prioritization to present results from the studies with highest internal validity, the 16 noncomparative studies are relegated to Appendix B. The 28 comparative studies were published between 1985 and 2011, with the majority (68%) published after 2000 (Table 1). Most studies were published in peer-reviewed journals (86%), three were reports [34, 40, 41], and there was one conference abstract included [38]. Three quarters of the studies were judged to be of low methodological study quality, 14% of moderate quality, and 11% of high methodological quality. It was a strength that in all studies, except 5 registry studies [18, 36–38, 45], the authors explained that the nonexposed group (non-FGM/C) was selected from the same population as the exposed group (FGM/C). When groups being compared are selected from different populations it offers less confidence in the effect estimates. Unfortunately, most of the studies failed to show that the groups were comparable with respect to important background factors and whether the person who assessed the outcome was blind to whether participants were exposed (had FGM/C) or not. Three of the comparative studies were Demographic and Health Surveys (DHS), which are nationally-representative household surveys [40, 41, 46], 1 study was based on a representative survey of households in Egypt [47], while the majority (68%) was nonrandom, clinical, or hospital-based studies. The representative surveys showed a self-reported prevalence of problems during delivery of 3%–40% across types of FGM/C [40, 41].

Overall, the 28 included comparative studies involved almost 3 million women (2,974 569; range 114–2,18 million). Most of the studies (71%) were conducted in a country in Africa, but 8 studies were carried out in a country in Europe or North America, and 1 study was from Saudi Arabia. Across the studies, the women's mean age was 26. With respect to FGM/C characteristics, 5 registry studies [18, 36–38, 45] appeared to include only women with FGM/C type III. In each of the remaining 16 studies that explained which type of FGM/C the women had been subjected to, there was a mix of genital alterations, but the most common type of FGM/C was type III (ca 41% of the women). About 31% of the women were described as having FGM/C type II and 22% as type I. In the majority of the studies (64%), the women were examined gynaecologically, generally both to confirm whether or not they had been subjected to FGM/C and to which type of FGM/C they had been subjected. Data regarding age of cutting and who performed the procedure were scarce, but when such data were available, typically, the women self-reported the FGM/C procedure to early childhood (mean age ca 7) and to a traditional circumciser. The most frequently reported outcomes were cesarean section, episiotomy, and obstetric tears. The majority of the studies (57%) had clinically measured obstetric outcomes, but 33% relied on women's self-report, and 2 studies did not explain how the outcomes were ascertained [30, 38].

### 3.2. Synthesis of Data

Several studies reported the same outcome and were sufficiently similar to warrant pooling of effect sizes in meta-analyses. Altogether we could conduct meta-analyses for the outcomes prolonged labor, obstetric tears/lacerations, caesarean section, episiotomy, instrumental delivery, obstetric/postpartum hemorrhage, and difficult labor/dystocia. The outcome data from each study are shown with the meta-analyses or in tables. Unless otherwise noted, all data are published data, and as shown in the figures, the meta-analyses evidenced large, unexplained heterogeneity across studies.

As a reiteration of the preceding section and a preface to the results and discussion in the latter part of the article, we stress that when it comes to establishing a causal relationship between exposure to a procedure such as FGM/C and an outcome, evidence based on observational studies will be appreciably weaker (usually) than evidence from experimental studies (to prove cause and effect, association is not enough: all plausible alternative explanations must be ruled out. This is best achieved through controlled research designs, but also through strength of evidence of association and evidence of a dose-response gradient [63]). In this systematic review, all included studies were necessarily observational and the majority of the studies had methodological shortcomings. Using GRADE, we judged the quality of the evidence for all outcomes as “very low,” which is defined “we have very little confidence in the effect estimate: the true effect is likely to be substantially different from the estimate of effect” [27].

#### 3.2.1. Prolonged Labor

**Figure 2:**
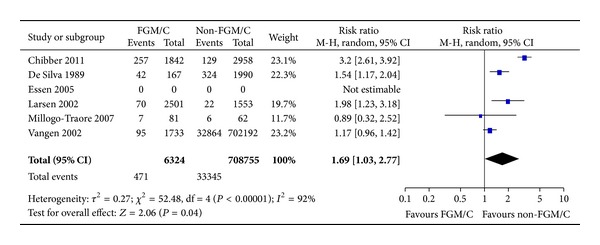
Forest plot, prolonged labor. Note: Sensitivity analyses for outcome (prolonged labor stage) and study type were not statistically significant. Data were missing in Essén et al. [36], and we did not succeed in obtaining data from the authors; thus, results from this study are not estimable.

Nine studies measured differences between women with FGM/C and women without FGM/C with respect to prolonged labor. We conducted meta-analysis for this outcome, pooling available data from five studies reporting a dichotomous measure of prolonged labor. Altogether, 715,079 women were included, of whom 6324 had FGM/C type I–IV. The outcome data are shown with the meta-analysis (Figure 2). Evident from the forest plot, there was a statistically significant difference between the two groups of women, favoring the non-FGM/C group (RR = 1.69, 95% CI = 1.03, 2.77). The absolute risk difference was 3 more cases of prolonged labor among women with FGM/C (95% CI = 0–8 more per 100 women).

Four studies presented prolonged labor as a continuous outcome, but essential data were missing to calculate mean difference, and/or the outcomes were not sufficiently similar to warrant meta-analyses. As shown in Table 2, the duration of labor for women with FGM/C versus non-FGM/C women varied across the studies with no observable pattern.

#### 3.2.2. Obstetric Tears/Lacerations

**Figure 3:**
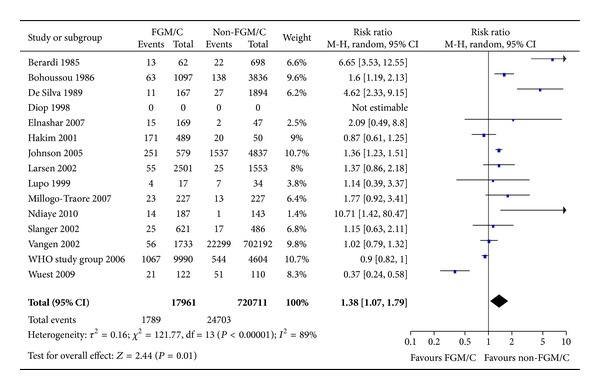
Forest plot, obstetric tears/lacerations. Note: Sensitivity analyses for outcome (degree of tears) and study type were not statistically significant. Data were missing in Diop et al. [34], and we did not succeed in obtaining data from the authors; thus, results from this study are not estimable. WHO study group [19]: unpublished data.

Regarding the outcome obstetric lacerations, we found a significant effect (15 studies, RR = 1.38, 95% CI = 1.07, 1.79; Figure 3). The absolute risk difference was 1,5 more cases of lacerations among women with FGM/C (95% CI = 0–3 more per 100 women). In total, 738,672 women were included, and 17,961 had been subjected to FGM/C of type I–IV.

#### 3.2.3. Caesarean Section

**Figure 4:**
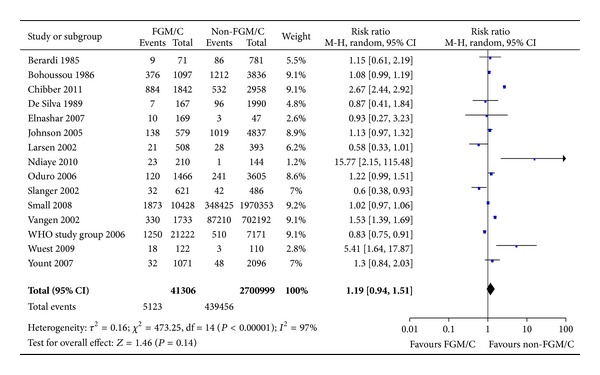
Forest plot, cesarean section. Note: Sensitivity analyses for study type were not statistically significant.

A total of 15 studies reported the prevalence of cesarean section for women with FGM/C compared to women without. There were 2.7 million women included in the meta-analysis, of whom 41,306 had FGM/C type I–IV. As evident from the forest plot (Figure 4), no statistically significant difference for cesarean section was found (RR = 1.19, 95% CI = 0.94, 1.51). The absolute risk difference was 8 more cases of caesarean section among women with FGM/C (95% CI = 0–18 more per 100 women).

#### 3.2.4. Episiotomy

**Figure 5:**
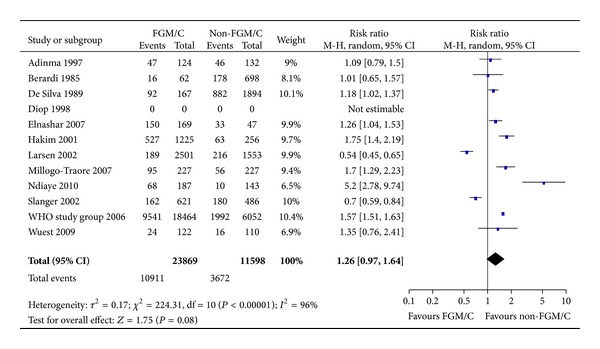
Forest plot, episiotomy. Note: Sensitivity analyses for parity were not statistically significant. Data were missing in Diop et al. [34], and we did not succeed in obtaining data from the authors; thus, results from this study are not estimable. WHO study group [19]: unpublished data.

We also conducted meta-analysis of the outcome episiotomy (Figure 5). In total, 35,467 women were included, and 23,869 (67%) had FGM/C type I–IV. No significant effect for this outcome was found (11 studies, RR = 1.26, 95% CI = 0.97, 1.64). The absolute risk difference was 6 more cases of episiotomy among women with FGM/C (95% CI = 1 fewer to 14 more per 100 women).

#### 3.2.5. Instrumental Delivery

**Figure 6:**
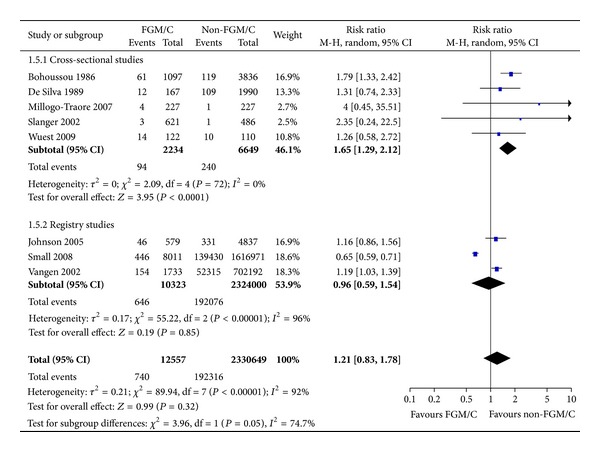
Forest plot, instrumental delivery.

Eight studies, including 3 registry studies, reported on instrumental delivery (2.3 million women, of whom 12,557 had FGM/C type I–IV). In the studies, instrumental delivery was described as ventouse, forceps, operative, or instrumental delivery. These studies' results are presented in Figure 6 with the results of the meta-analysis. Sensitivity analyses were conducted for study type and showed a significant difference between the cross-sectional studies and the registry studies. The pooled result from the cross-sectional studies where the study participants were selected from the same population shows that women with FGM/C are more likely than women with no FGM/C to require instrumental delivery (RR = 1.65, 95% CI = 1.29, 2.12). The absolute risk difference was 2 more cases of instrumental delivery among women with FGM/C (95% CI = 1–4 more per 100 women). Conversely, registry studies, comparing Somali-born women (likely FGM/C type III) and Western-born women without FGM/C showed no statistically significant difference between the two groups of women with respect to instrumental delivery (RR = 0.96, 95% CI = 0.59, 1.54). There was large, unexplained heterogeneity across the registry studies, but not the cross-sectional studies.

#### 3.2.6. Obstetric/Postpartum Hemorrhage

**Figure 7:**
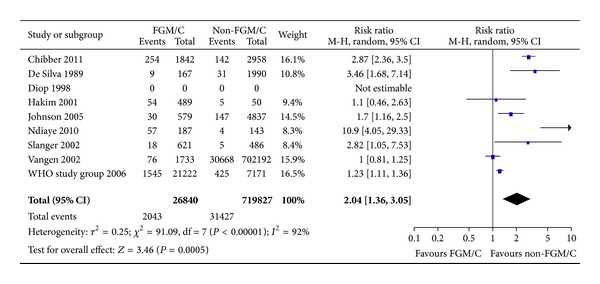
Forest plot, obstetric/post-partum hemorrhage. Note: Sensitivity analyses for outcome (definition) and study type were not statistically significant. Data were missing in Diop et al. [34], and we did not succeed in obtaining data from the authors; thus, results from this study are not estimable.

Ten included studies measured differences between women with FGM/C and without FGM/C with respect to obstetric hemorrhage. Nine of the studies measured this as a dichotomous outcome and were sufficiently similar to warrant pooling in meta-analysis. There were 746,667 women included, and women with FGM/C type I–IV made up 3.7%. As shown in Figure 7, there was a significant effect (RR = 2.04, 95% CI = 1.36, 3.05). The absolute risk difference was 5 more cases of obstetric hemorrhage among women with FGM/C (95% CI = 2–9 more per 100 women).

One study [20] used a continuous measure for maternal blood loss during labor, measured as mL blood loss, which ranged from 100 to 3500 mL among the patients (Table 2). Women who had gone through FGM/C experienced a median of 50 mL blood loss more than non-FGM/C women during labor.

#### 3.2.7. Difficult Labor/Dystocia

**Figure 8:**
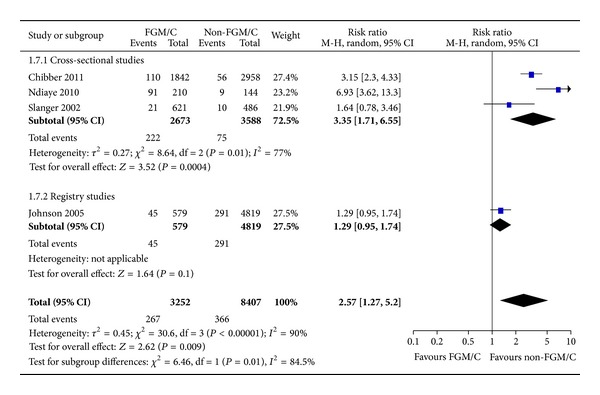
Forest plot, difficult delivery.

Regarding the outcome difficult labor, seven studies examined this outcome among women with FGM/C and women without FGM/C. In total, there were 11,659 women, of whom 3252 had FGM/C type I–IV. The sensitivity analysis demonstrated a significant difference between cross-sectional, Africa-based studies and the registry study. The pooled result from cross-sectional studies where the participants were selected from the same population shows that women with FGM/C are more likely than women with no FGM/C to experience difficult labor (Figure 8, RR = 3.35, 95% CI = 1.71, 6.55). The absolute risk difference was 5 more cases of difficult labor among women with FGM/C (95% CI = 1–12 more per 100 women). Conversely, the registry study, comparing Somali-born women (likely FGM/C type III) and US-born women showed no statistically significant difference between the two groups of women regarding difficult labor (Figure 8, RR = 1.29, 95% CI = 0.95, 1.74).

## 4. Discussion

This systematic review aimed to answer a question on the minds of many women, health care providers, researchers, activists, and policy makers: what additional risks does a woman who has undergone FGM/C assume related to delivery, compared to a woman without FGM/C? The low quality of the of body of evidence does not allow for obstetric complications to be causally attributed to FGM/C, but our results from seven meta-analyses support the claim that FGM/C exerts a negative impact on a range of obstetric events. The estimates for prolonged labor, obstetric lacerations, instrumental delivery, obstetric hemorrhage, and difficult delivery demonstrate disparities in obstetric outcomes for women with FGM/C relative to women who have not been subjected to FGM/C. 

### 4.1. Discussion of Main Results

The results showed that women with FGM/C were 3.3 times more likely to experience difficult labor and twice as likely to experience obstetric hemorrhage compared to women without FGM/C. In absolute terms, the risk difference was on average 5 additional cases of difficult labor and 5 additional cases of obstetric hemorrhage among women with FGM/C per 100 women. Since the studies in the meta-analyses included women with various types of FGM/C, genital alteration of any type seems to be associated with obstetric complications, although the mechanism by which FGM/C may cause problems during delivery remains unresolved. However, FGM/C is a physiologically plausible explanation for the increased risk of obstetric lacerations and hemorrhage in particular, because of the inelasticity of scar tissue from FGM/C. Further, inelastic scar tissue may contribute to obstructions, which may prolong labor. Browning et al. [31] explain that increased scarring around the introitus from more invasive FGM/C can cause a delay in the second stage of labor. In turn, a longer second stage of labor could underlie the increased risk of perineal lacerations and hemorrhage among women with FGM/C identified in our study. Moreover, results of the meta-analysis for episiotomy showed no statistically significant difference between women with and without FGM/C. It is possible that lack of episiotomy contributes to the occurrence of obstetric lacerations, as suggested by experts [64]. It follows that episiotomy may be justifiable among women with FGM/C, particularly those with type II and III, in order to limit the degree of perineal laceration and bleeding that may occur in these women.

In Africa, where FGM/C typically is practiced, maternal morbidity and mortality rates are much higher than in more developed regions [65, 66], with haemorrhage as the leading cause of maternal mortality [67]. FGM/C seems to be an underlying factor that increases the risk of such complications, and it may lead to additional cases of adverse maternal outcomes. Moreover, we did not assess outcomes related to the child, but several studies have documented an increased risk of fetal distress in women with FGM/C [14, 32]. For example, the WHO study group [19] results indicated that FGM/C could lead to 1-2 additional perinatal deaths per 100 deliveries. The societies where FGM/C is widely practiced are generally pronatalist and value large families. Larsen and Okonofua [16] explain that in these areas, motherhood is a principal source of support, status, and security. In this context, the now sounder understanding of anticipated obstetric improvements with the halting of FGM/C can be used as a strategy for campaigning against the practice, for example, by centering the message on safe delivery. The obstetric consequences from FGM/C can no longer be ignored, and the results of this systematic review provide another strong argument for the provision of culturally grounded knowledge that can contribute to public awareness about FGM/C. It is possible that once greater awareness exists of the increased risk of adverse labor outcomes following FGM/C, the practice may be less firmly supported. The results should also be included in the education and training of not just those involved in interventions against the practice but also health care providers and in clinical guidelines for managing women who have undergone FGM/C.

In a multistage modeling analysis, which was based on the 2006 WHO study in which about 28,000 women and their newborns were monitored for adverse health outcomes at obstetric centers in six countries, the costs associated with obstetric complications related to FGM/C were estimated. The researchers calculated that compared to a 15-year-old who does not undergo FGM/C, the average 15-year-old who undergoes any type of FGM/C loses 0.07 of a year of life and generates $1.71 (international dollars) of associated medical costs over her lifetime. The costs for a woman with FGM/C type III were considerably greater [68]. While the health and financial loss on an individual level may seem small, overall, the estimated national costs ranged from 0.1% to 1% of government health spending on care for FGM/C related problems [68]. Presumably, obstetric complications, such as the ones we examined in this systematic review, account for only a small portion of the overall health impact of FGM/C on the affected woman and in a population. By extension, the financial costs of obstetric complications are merely one among many possible costs associated with the practice.

Experiencing a birth-related complication inflicts distress not just on the individual woman, but potentially also her baby, partner, family, and there are economic burdens imposed on the health system from providing care for these women. Writers such as Mawad and Hassanein [69] state that with careful planning, good antenatal, intrapartum, and postpartum care, most obstetric problems associated with FGM/C can be avoided. The claim itself is questionable from medical and research standpoints, and unfortunately, in some high FGM/C prevalence areas health care resources are often unavailable and public health services malfunctioning, which means that a considerable number of women who deliver within health services are not attended by qualified health personnel [70]. In fact, many women give birth at home [70, 71] and in eastern and southern Africa, half of all births occur without the support of a skilled birth attendant [72]. Moreover, our systematic review results based on registry studies taking place in western countries—where women are likely to receive good antenatal, intrapartum, and postpartum care—showed that for all outcomes, except instrumental delivery, women with FGM/C fared worse than women without FGM/C. This strengthens the argument for a true association between FGM/C and obstetric complications.

With regards to instrumental delivery, the meta-analyses results for registry studies comparing Somali-born women and western-born women showed a lower, nonsignificant risk among Somali-born women, who likely had FGM/C type III. This could be related to Somali women holding culturally anchored beliefs about natural childbirth that lead to reluctance to accept obstetric interventions. According to qualitative studies, Somali women in diaspora express anxiety about childbirth interventions, a general dislike of interference in the birth process, and difficulties in communication with caregivers [73–75]. Related to the result of instrumental delivery, we found no statistically significant excess of experiencing cesarean section and episiotomy among women with FGM/C. However, the direction of effect across studies, particularly for episiotomy, certainly seemed to favor women not having FGM/C.

### 4.2. Strengths and Limitations

Some caution is warranted in interpreting these meta-analytic results. While the results rest on a methodology that meets the PRISMA criteria for systematic reviews [12], our search was completed in January 2012, and newer studies may exist. Despite a comprehensive search strategy, publication bias may be present with the likeliest scenario being that the results are biased to the positive. We failed to obtain 13 relevant records in full text as well as primary data from 3 studies which potentially could have been included in meta-analyses [15, 34, 36]. On the other hand, we received and included unpublished data from the WHO study group on female genital mutilation and obstetric outcome [19]. Using GRADE, we assessed the quality of the evidence for all outcomes as being too low to warrant conclusions about a causal relationship between FGM/C and obstetric complications. This was largely due to not only the weaknesses of the observational design of all included studies—which illustrates the practical barriers to health outcomes research related to FGM/C—but also inconsistencies in results and estimate imprecision. Despite the large sample sizes for all of the pooled analyses (range 11,659–2.7 million) the confidence intervals for many of the effect estimates remained wide. The inclusion of missed studies and future outcome research could narrow the confidence intervals, but for most outcomes only very large studies would alter the direction of effect.

Measurement of “exposure” to FGM/C can be a methodological challenge. However, we applied the WHO classification system for FGM/C type I through IV [3], and a similar classification system was applied in most of the included studies. Further, 69% of the comparative studies based classification and exposure on gynaecological examination. It was also a strength that measurement of the majority of the obstetric outcomes was clinically based. On the other hand, there was a lack of a unified approach and standardized definitions to measure common outcomes such as prolonged labor. When definitions were missing we relied on the terminology and categories used in the publications, but we could not always be sure that similarly labeled outcomes were identically defined and measured in each study. In a broader perspective, this may not be a serious limitation as the crucial question is whether the risk of obstetric complications, in the general case, not only specific to certain outcomes, is greater among women with FGM/C than women not subjected to the procedure.

## 5. Conclusions

The need for synthesized scientific research to specify the health sequelae of FGM/C, obstetric events in particular, motivated this systematic review. While the low quality of the body of evidence means that it is unclear whether the documented association of FGM/C with obstetric complications reflects true causality, the evidence base shows that deliveries to women who have undergone FGM/C are more likely to be complicated compared to deliveries to women who have not been subjected to the practice.

Consonant with other review findings [7, 8, 10], our systematic review results show no indication of there being obstetric benefits to FGM/C. Rather, today's best available evidence documents a significantly greater risk for prolonged labor, obstetric lacerations, instrumental delivery, obstetric hemorrhage, and difficult delivery among women with FGM/C relative to women with no FGM/C and no significant difference in risk with respect to cesarean section and episiotomy. The exact size of the greater obstetric risk from FGM/C is unclear, but the increased risk of harm is unmistakable, such that the data clarify the obstetric improvements that may be anticipated with discontinuing FGM/C. Given the volume of data and practical difficulties with health outcomes research of more valid study designs related to FGM/C, it is questionable whether intensified research efforts would change the present findings. From a women's health standpoint, irrespective of the exact size of the greater risk from FGM/C, the increase in obstetric suffering and morbidity is too high to justify continuing the practice. If further research on the association between FGM/C and obstetric outcomes is considered ethically and financially justified, such studies should be based on the best possible and practically feasible methodological study design, which for FGM/C obstetrics outcome research is case-control studies. Additional cross-sectional studies would possibly narrow the confidence intervals, but it is unlikely that the direction of the estimates of obstetric outcomes would change. Lastly, any future research should be based on a methodology that ensures representativeness and equivalency between exposed and unexposed groups of women, and that applies standardized definitions and clinical measures for exposure as well as outcomes.

## Figures and Tables

**Table 1:** Included comparative studies reporting on obstetric events (*n* = 28).

Author, year	Method study quality	Population total *N* (cut)	Country/origin	Age	FGM/C characteristics	Outcomes (self-report or clinical verification)
Adinma 1997 [28]	Low	*N* = 256 (124)	Nigeria	16–40	Type: 22% TI, 78% TII (gyn exam) Age cut/by: 97% in childhood/not stated	Episiotomy (self-report)
Berardi et al. 1985 [29]	Low	*N* = 852 (71)	France	Not stated	Type: 100% TII (gyn exam) Age cut/by: not stated	Tears; cesarean section; episiotomy (clinical)
Bohoussou et al. 1986 [30]	Low	*N* = 4935 (1099)	Ivory Coast	Not stated	Type: 29% TI, 73% TII (gyn exam) Age cut/by: not stated	Prolonged labor; tears; cesarean section; episiotomy; instrumental delivery (not stated)
Browning et al. 2010 [31]	High	*N* = 492 (255)	Ethiopia	Mean 28.5	Type: 100% TI and TII (gyn exam) Age cut/by: not stated	Prolonged labor (clinical)
Chibber et al. 2011 [32]	Low	*N* = 4800 (1842)	Not stated	Not stated	Type: “type I to III most common” (gyn exam) Age cut/by: not stated	Prolonged labor; cesarean section; hemorrhage (clinical)
De Silva 1989 [33]	Low	*N* = 2157 (167)	Saudi Arabia	Not stated	Type: 9% TI, 34% TII, 32% TIII (gyn exam) Age cut/by: not stated	Prolonged labor; tears; cesarean section; episiotomy; instrumental delivery; hemorrhage (clinical)
Diop et al. 1998 [34]	Low	*N* = 5390 (4359)	Mali	Mean 27.0	Type: 21% TI, 73% TII, 6% TIII (gyn exam) Age cut/by: not stated	Tears; episiotomy; hemorrhage (clinical)
Elnashar and Abdelhady 2007 [35]	Low	*N* = 264 (200)	Egypt	Not stated	Type: “circumcised” (self-report) Age cut/by: not stated	Tears; cesarean section; episiotomy (self-report)
Essén et al. 2005 [36]	Moderate	*N* = 2554 (68)	Sweden	Not stated	Type: most TIII (gyn exam) Age cut/by: not stated	Prolonged labor (clinical)
Hakim 2001 [14]	Low	*N* = 1481 (1225)	Ethiopia	Mean 25.9	Type: 12% TI, 85% TII, 3% TIII (not stated) Age cut/by: not stated	Prolonged labor; tears; episiotomy; hemorrhage (clinical)
Johnson 2005 [37]	Low	*N* = 5416 (579)	USA	Most 20–34	Type: most likely type III (assumed, unverified). Age cut/by: not stated	Tears; cesarean section; instrumental delivery; hemorrhage (clinical)
Jones et al. 1999 [15]^b^	Low	*N* = 1920 (1787)	Burkina Faso	Mean 26.6	Type: 56% TI, 39% TII, 5% TIII (gyn exam) Age cut/by median 9.5 yrs/not stated	Difficult labor (self-report)
Jones et al. 1999 [15]^b^	Moderate	*N* = 5337 (5017)	Mali	Mean 25.0	Type: 21% TI, 74% TII, 5% TIII (gyn exam) Age cut/by: not stated	Difficult labor (clinical)
Larsen and Okonofua 2002 [16]	Low	*N* = 1836 (1009)	Nigeria	15–49	Type: 71% TI, 25% TII, 3% TIII, 1% TIV (gyn exam). Age cut/by: not stated	Prolonged labor; tears; cesarean section; episiotomy (self-report)
Lupo and Marcotte 1999 [38]	Low	*N* = 114 (38)	USA	Not stated	Type: “female circumcision” (not stated) Age cut/by: not stated	Tears (not stated)
Millogo-Traore et al. 2007 [39]	Low	*N* = 454 (227)	Burkina Faso	Median 25	Type: 28% TI, 69% TII, 3% TIII (gyn exam) Age cut/by: not stated	Prolonged labor; tears; episiotomy; instrumental delivery (clinical)
National Statistics Office 1995 [40]	Low	*N* = 4775^a^	Eritrea	15–49	Type: 62% TI, 4% TII, 34% TIII (self-report) Age cut/by: 60% ≤5 yrs/91% tc	Problems during delivery (self-report)
NSEO 2003 [41]	Low	*N* = 7765^a^	Eritrea	15–49	Type: 4% TI-II, 39% TIII, 46% TIV (self-report) Age cut/by: 62% ≤1 yr/84% tc	Problems during delivery (self-report)
Ndlaye et al. 2010 [42]	Low	*N* = 354 (210)	Burkina Faso	Mean 24.0	Type: 47% TI, 47% TII, 6% TIII (gyn exam) Age cut/by: not stated	Tears; cesarean section; episiotomy; hemorrhage (clinical)
Oduro et al. 2006 [43]	High	*N* = 5071 (1466)	Ghana	Mean 25.8	Type: “type II is the commonest form” (gyn exam). Age cut/by: not stated	Cesarean section (clinical)
Orji and Babalola 2006 [17]	Low	*N* = 500 (423)	Nigeria	Mean 27.5	Type: 87% TI, 13% TII (gyn exam) Age cut/by: 95% cut in childhood/80% tc, 14% hcp	Cesarean section; episiotomy (self-report)
Slanger et al. 2002 [44]	Moderate	*N* = 1107 (621)	Nigeria	Mean 33.7	Type: 72% TI, 24% TII, 4% TIII + IV (gyn exam), Age cut/by: 95% cut in childhood/80% tc, 14% hcp	Tears; cesarean section; episiotomy; instrumental delivery; hemorrhage; fever (self-report)
Small et al. 2008 [45]	Low	*N* = 2179322 (10431)	Multiple^c^	Most 20–34	Type: most likely type III (assumed, unverified). Age cut/by: not stated	Cesarean section; instrumental delivery (clinical)
Vangen et al. 2002 [18]	Low	*N* = 703925 (1733)	Norway	Not stated	Type: most likely type III (assumed, unverified). Age cut/by: not stated	Prolonged labor; tears; cesarean section; hemorrhage (clinical)
WHO study group 2006 [19]	High	*N* = 28393 (21222)	Multiple^d^	Mean 26.3	Type: 32% TI, 37% TII, 31% TIII (gyn exam) Age cut/by: not stated	Tears; cesarean section; episiotomy; hemorrhage (clinical)
Wuest et al. 2009 [20]	Low	*N* = 232 (122)	Switzerland	Mean 28.0	Type: 17% TI, 24% TII, 48% TIII, 11% TIV (gyn exam). Age cut/by: not stated	Prolonged labor; tears; cesarean section; episiotomy; instrumental delivery; hemorrhage (clinical)
Yount and Abraham 2007 [46]	Moderate	*N* = 3167 (1071)	Kenya	15–49	Type: “had undergone FGC” (self-report) Age cut/by: not stated	Cesarean section (self-report)
Yount and Carrera 2006 [47]	Low	*N* = 1700^a^	Egypt	17–55	Type: 4% TI, 73% TII, 23% TIV (self-report) Age cut/by: mode 9-10 yrs/93% tc, 4% hcp	Pregnancy loss (self-report): 39% TI, 42% TII, 43% TIV

Legend: Method: Methodological; TI: FGM/C type I; TII: FGM/C type II; TIII: FGM/C type III; TIV: FGM/C type IV; gyn exam: FGM/C status verified through gynecological examination; self-report: FGM/C status based on self-report; hcp: health care provider; tc: traditional circumciser; ^a^different types of FGM/C were compared; ^b^Jones et al. 1999 [15] consists of two studies, reported in same publication; ^c^Australia, Belgium, Canada, Finland, Norway, and Sweden; ^d^Burkina Faso, Ghana, Kenya, Nigeria, Sudan, and Senegal.

**Table 2:** Continuous study outcomes and effect estimates.

Author, year	Outcome	FGM/C group	Non-FGM/C group	Results Mean diff (95% CI)
Browning et al. 2010 [31]	Days in labor	3.1 (1.7) days	2.8 (1.5) days	0.30 (0.02, 0.58)*

Essén et al. 2005 [36]	Duration of labor stage 2	35 min^a^	53 min	—

Hakim 2001 [14]	Duration of labor stage 1	11.8 (4.7) hrs (708 min)	11.6 (2.2) hrs (696 min)	0.20 (−0.54, 0.94)
	Duration of labor stage 2	41.5 (13.3) min	40.1 (3.2) min	1.40 (−0.08, 2.88)
	Duration of labor stage 3	11.0 (4.0) min	11.1 (4.5) min	−0.10 (−1.40, 1.20)

Wuest et al. 2009 [20]	Duration of labor stage 1	220 min^a^	300 min	—
	Duration of labor stage 2	39 min	45 min	—
	Maternal blood loss	400 mL (range 200–1000)	350 mL (range 100–3500)	−50 (*P* = 0.81)

Legend: Mean diff: mean difference; ^a^Essén et al. 2005 [36] and Wuest et al. 2009 [20] reported duration of labor as median minutes (not mean); *statistically significant.

**Table 3:** 

Author, year	Study design	Method study quality	Population, country	Outcomes (self-report or clinical verification)
Abor 2006 [48]	Cross-sectional	Low	*N* = 34, Ghana	Cesarean section (17%); episiotomy (29%); instrumental delivery (8%) (self-report)
Akotionga et al. 2001 [55]	Case series	High	*N* = 49, Burkina Faso	Difficult delivery (13%) (clinical)
Al-Hussaini 2003 [49]	Cross-sectional	Moderate	*N* = 254, Egypt	Tears (2%); cesarean section (17%); episiotomy (95%) (clinical)
Awuah 2008 [56]	Case series	Low	*N* = 70, Ghana	Prolonged labor stage 1 (37%); prolonged labor stage 2 (9%); massive tears (23%; damage to rectal wall (13%); episiotomy (14%); hemorrhage (24%) (self-report)
Bayoudh et al. 1995 [50]	Cross-sectional	Low	*N* = 300, Somalia	Episiotomy (3%) (self-report)
Bonessio et al. 2001 [57]	Case series	Low	*N* = 9, Italy	Prolonged labor (25%); cesarean section (25%) (clinical)
Chalmers and Hashi 2000 [51]	Cross-sectional	Low	*N* = 432, Canada	Cesarean section (51%); vacuum extraction (7%); forceps (3%) (self-report)
Dörflinger et al. 2000 [58]	Case series	Low	*N* = 39, Sudan	Prolonged labor stage 1 (7%); prolonged labor stage 2 (24%); tears (7%); hemorrhage (14%) (clinical)
Litorp et al. 2008 [52]	Cross-sectional	Low	*N* = 40, Sweden	Obstetric difficulties (self-report)
Mccaffrey 1995 [53]	Cross-sectional	Low	*N* = 50, England	Tears (100%); cesarean section (26%); Instrumental delivery (13%) (clinical)
McSwiney and Saunders 1992 [59]	Case report	NA	*N* = 1, England	Tears led to rapid hemorrhage (clinical)
Ndamobissi et al. 1995 [54]	Cross-sectional	High	*N* = 2555, Central African Republic	Obstetric complications (self-report)
Osifo and Evbuomwan 2009 [6]	Case series	High	*N* = 51, Nigeria	Tears (4%) led to uncontrolled bleeding (clinical)
Philp 1927 [60]	Case report	NA	*N* = 1, Kenya	Death in childbirth (clinical)
Preston 1937 [61]	Case report	NA	*N* = 1, Kenya	Birth per rectum (clinical)
Pritchard 1969 [62]	Case report	NA	*N* = 3, England	Dystocia (clinical)

Legend: Method.: methodological; NA: not applicable, we did not assess methodological study quality of case reports.

## Appendices

### A. MEDLINE Search

*Database*. Ovid MEDLINE(R) In-Process & Other Non-Indexed Citations and Ovid MEDLINE(R) 1946 to Present (1946 to January 19, 2012).

*Search*

1. Circumcision, Female/
2. ((female$ or wom#n or girl$1) adj3 (mutilation$ or circumcis$ or cutting$)).tw.
3. “fgm/c”.tw.
4. ((removal$ or alteration$ or excision$) adj6 female genital$).tw.
5. pharaonic circumcision$.tw.
6. sunna.tw.
7. (clitoridectom$ or clitorectom$).tw.
8. (infibulat$ or reinfibulat$ or deinfibulat$).tw.
9. or/1–8.

### B. Noncomparative Studies

See Table 3.

## References

[1] Monjok E, Essien EJ, Holmes L (2007). Female genital mutilation: potential for HIV transmission in sub-Saharan Africa and prospect for epidemiologic investigation and intervention. *African Journal of Reproductive Health*.

[2] WHO (2011). *An Update on WHO’s Work on Female Genital Mutilation (FGM), Progress Report*.

[3] WHO (2008). *Eliminating Female Genital Mutilation: An Interagency Statement*.

[4] Berg RC, Denison E (2012). A tradition in transition: factors perpetuating and hindering the continuance of female genital mutilation/cutting (FGM/C) summarized in a systematic review. *Health Care for Women International*.

[5] Anuforo PO, Oyedele L, Pacquiao DF (2004). Comparative study of meanings, beliefs, and practices of female circumcision among three Nigerian tribes in the United States and Nigeria. *Journal of Transcultural Nursing*.

[6] Osifo DO, Evbuomwan I (2009). Female genital mutilation among Edo people: the complications and pattern of presentation at a pediatric surgery unit, Benin City. *African Journal of Reproductive Health*.

[7] Obermeyer CM (1999). Female genital surgeries: the known, the unknown, and the unknowable. *Medical Anthropology Quarterly*.

[8] Obermeyer CM (2005). The consequences of female circumcision for health and sexuality: an update on the evidence. *Culture, Health and Sexuality*.

[9] Berg RC, Denison E (2012). Does female genital mutilation/cutting (FGM/C) affect women’s sexual functioning? A systematic review of the sexual consequences of FGM/C. *Sexuality Research and Social Policy*.

[10] WHO (2000). *A Systematic Review of the Health Complications of Female Genital Mutilation Including Sequelae in Childbirth*.

[11] Higgins JPT, Green S (2011). *Cochrane Handbook for Systematic Reviews of Interventions, Version 5.1.0*.

[12] Moher D, Liberati A, Tetzlaff J, Altman DG, The PRISMA Group (2009). Preferred reporting items for systematic reviews and meta-analyses: the PRISMA statement. *PLoS Medicine*.

[13] Petticrew M, Roberts H (2006). *Systematic Reviews in the Social Sciences. A Practical Guide*.

[14] Hakim LY (2001). Impact of female genital mutilation on maternal and neonatal outcomes during parturition. *East African Medical Journal*.

[15] Jones H, Diop N, Askew I, Kaboré I (1999). Female genital cutting practices in Burkina Faso and Mali and their negative health outcomes. *Studies in Family Planning*.

[16] Larsen U, Okonofua FE (2002). Female circumcision and obstetric complications. *International Journal of Gynecology and Obstetrics*.

[17] Orji EO, Babalola A (2006). Correlates of female genital mutilation and its impact on safe motherhood. *Journal of the Turkish German Gynecology Association*.

[18] Vangen S, Stoltenberg C, Johansen REB, Sundby J, Stray-Pedersen B (2002). Perinatal complications among ethnic Somalis in Norway. *Acta Obstetricia et Gynecologica Scandinavica*.

[19] WHO Study Group on Female Genital Mutilation and Obstetric Outcome (2006). Female genital mutilation and obstetric outcome: WHO collaborative prospective study in six African countries. *The Lancet*.

[20] Wuest S, Raio L, Wyssmueller D (2009). Effects of female genital mutilation on birth outcomes in Switzerland. *BJOG: An International Journal of Obstetrics and Gynaecology*.

[21] The Public Policy Advisory Network on Female Genital Surgeries in Africa (2012). Seven things to know about female genital surgeries in Africa. *Hastings Center Report*.

[22] Berg RC, Underland V Obstetric consequences of female genital mutilation/cutting (FGM/C).

[23] Nasjonalt kunnskapssenter for helsetjenesten (2011). Slik oppsummerer vi forskning. *Håndbok for Nasjonalt kunnskapssenter for helsetjenesten*.

[24] Review Manager (RevMan) [Computer program] (2012). *Version 5.2. Copenhagen: The Nordic Cochrane Centre*.

[25] Brozek J, Oxman A, Schünemann H

[26] Guyatt G, Oxman AD, Akl EA (2011). GRADE guidelines: 1. Introduction—GRADE evidence profiles and summary of findings tables. *Journal of Clinical Epidemiology*.

[27] Balshem H, Helfand M, Schünemann HJ (2011). GRADE guidelines: 3. Rating the quality of evidence. *Journal of Clinical Epidemiology*.

[28] Adinma JI (1997). Current status of female circumcision among Nigerian Igbos. *West African Journal of Medicine*.

[29] Berardi JC, Teillet JF, Godard J, Laloux V, Allane P, Franjou MH (1985). Consequences obstetricales de l'excision feminine. Etude chez 71 femmes africaines excisees. *Journal de Gynecologie, Obstetrique et Biologie de la Reproduction*.

[30] Bohoussou KM, Anongba S, Djanhan Y, Bonis S, Ble B, Sangaret MA (1986). Complications gynecologiques, medicales et obstetricales de l’excision rituelle. *African Medicine*.

[31] Browning A, Allsworth JE, Wall LL (2010). The relationship between female genital cutting and obstetric fistulae. *Obstetrics and Gynecology*.

[32] Chibber R, El-Saleh E, El Harmi J (2011). Female circumcision: obstetrical and psychological sequelae continues unabated in the 21st century. *Journal of Maternal-Fetal and Neonatal Medicine*.

[33] de Silva S (1989). Obstetric sequelae of female circumcision. *European Journal of Obstetrics Gynecology and Reproductive Biology*.

[34] Diop N, Sangaré M, Tandia F, Touré K (1998). *Study of the Effectiveness of Training Malian Social and Health Agents in Female Genital Cutting Issues and in Educating Their Clients*.

[35] Elnashar A, Abdelhady R (2007). The impact of female genital cutting on health of newly married women. *International Journal of Gynecology and Obstetrics*.

[36] Essén B, Sjöberg NO, Gudmundsson S, Östergren PO, Lindqvist PG (2005). No association between female circumcision and prolonged labour: a case control study of immigrant women giving birth in Sweden. *European Journal of Obstetrics Gynecology and Reproductive Biology*.

[37] Johnson EB, Reed SD, Hitti J, Batra M (2005). Increased risk of adverse pregnancy outcome among Somali immigrants in Washington state. *The American Journal of Obstetrics and Gynecology*.

[38] Lupo V, Marcotte KL (1999). Obstetric complications of Somali female circumcision. *Obstetrics and Gynecology*.

[39] Millogo-Traore F, Kaba STA, Thieba B, Akotionga M, Lankoande J (2007). Maternal and foetal prognostic in excised women delivery. *Journal de Gynecologie Obstetrique et Biologie de la Reproduction*.

[40] National Statistics Office [Eritrea] (1995). *Eritrea Demographic and Health Survey*.

[41] National Statistics and Evaluation Office (NSEO) (2003). *Eritrea Demographic and Health Survey 2002*.

[42] Ndlaye P, Diongue M, Faye A, Ouedraogo D, Dia AT (2010). Female genital mutilation and complications in childbirth in the province of Gourma (Burkina Faso). *Sante Publique*.

[43] Oduro A, Ansah P, Hodgson A (2006). Trends in the prevalence of female genital mutilation and its effect on delivery outcomes in the Kassena-Nankana district of Northern Ghana. *Ghana Medical Journal*.

[44] Slanger TE, Snow RC, Okonofua FE (2002). The impact of female genital cutting on first delivery in Southwest Nigeria. *Studies in Family Planning*.

[45] Small R, Gagnon A, Gissler M (2008). Somali women and their pregnancy outcomes postmigration: data from six receiving countries. *BJOG: An International Journal of Obstetrics and Gynaecology*.

[46] Yount KM, Abraham BK (2007). Female genital cutting and HIV/AIDS among Kenyan Women. *Studies in Family Planning*.

[47] Yount KM, Carrera JS (2006). Female genital cutting and reproductive experience in Minya, Egypt. *Medical Anthropology Quarterly*.

[48] Abor PA (2006). Female genital mutilation: psychological and reproductive health consequences. The case of Kayoro traditional area in Ghana. *Gender and Behaviour*.

[49] Al-Hussaini TK (2003). Female genital cutting: types, motives and perineal damage in laboring Egyptian women. *Medical Principles and Practice*.

[50] Bayoudh F, Barrak S, Fredj NB, Allani R, Hamdi M (1995). Study of a common practice in Somalia: female circumcision. *Medecine Tropicale*.

[51] Chalmers B, Hashi KO (2000). 432 Somali women’s birth experiences in Canada after earlier female genital mutilation. *Birth*.

[52] Litorp H, Franck M, Almroth L (2008). Female genital mutilation among antenatal care and contraceptive advice attendees in Sweden. *Acta Obstetricia et Gynecologica Scandinavica*.

[53] Mccaffrey M (1995). Female genital mutilation: consequences for reproductive and sexual health. *Sexual and Marital Therapy*.

[54] Ndamobissi R, Mboup G, Nguélébé EO (1995). *Enquête Démographique et de Santé, République Centrafrieaine 1994-95*.

[55] Akotionga M, Traore O, Lakoande J, Kone B (2001). Sequelles genitales externes de l'excision au centre hospitalier national Yalgado Ouedraogo (CHN-YO): epidemiologie et traitement chirurgical. *Gynecologie Obstetrique Fertilite*.

[56] Awuah JB (2008). Female genital mutilation: a study in Aboabo, a suburb of Kumasi, Ghana. *West African Journal of Nursing*.

[57] Bonessio L, Bartucca B, Bertelli S, Morini F, Aleandri V, Spina V (2001). Mutilazioni genitali femminili: pazienti con esiti di FGM ricoverate nel policlinico “umberto I” di Roma: periodo 1985–1996. *Clinica Terapeutica*.

[58] Dörflinger A, Kuhn P, Dreher E (2000). Die zirkumzision der frau—(K)Ein rein Afrikanisches problem. *Geburtshilfe und Frauenheilkunde*.

[59] McSwiney MM, Saunders PR (1992). Female circumcision: a risk factor in postpartum haemorrhage. *Journal of Postgraduate Medicine*.

[60] Philp HRA (1927). Vescical fistula complicating labour. *Kenya and East African Medical Journal*.

[61] Preston PG (1937). A case of birth per rectum. *The East African Medical Journal*.

[62] Pritchard BJ (1969). Soft tissue dystocia in circumcised women. *Nursing Mirror and Midwives Journal*.

[63] Guyatt G, Meade M (2008). *User’s Guides to the Medical Literature. A Manual for Evidence-Based Clinical Practice*.

[64] Gibbs RS, Karlan BY, Hanley AF, Nygaard I (2008). *Danforth's Obstetrics and Gynecology*.

[65] Hogan MC, Foreman KJ, Naghavi M (2010). Maternal mortality for 181 countries, 1980–2008: a systematic analysis of progress towards millennium development goal 5. *The Lancet*.

[66] Lewis G (2004). *Beyond the Numbers: Reviewing Maternal Deaths and Complications to Make Pregnancy Safer*.

[67] Khan KS, Wojdyla D, Say L, Gülmezoglu AM, van Look PF (2006). WHO analysis of causes of maternal death: a systematic review. *The Lancet*.

[68] Bishai D, Bonnenfant Y, Darwish M (2010). Estimating the obstetric costs of female genital mutilation in six African countries. *Bulletin of the World Health Organization*.

[69] Mawad NM, Hassanein OM (1972). Maternity service in Khartoum civil hospital—part 1: general review. *Sudan Medical Journal*.

[70] Prual A, Bouvier-Colle MH, de Bernis L, Bréart G (2000). Severe maternal morbidity from direct obstetric causes in West Africa: incidence and case fatality rates. *Bulletin of the World Health Organization*.

[71] Ronsmans C, Etard JF, Walraven G (2003). Maternal mortality and access to obstetric services in West Africa. *Tropical Medicine and International Health*.

[72] UNICEF http://www.unicef.org/esaro/5479_maternal_newborn_health.html.

[73] Essén B, Johnsdotter S, Hovelius B (2000). Qualitative study of pregnancy and childbirth experiences in Somalian women resident in Sweden. *The British Journal of Obstetrics and Gynaecology*.

[74] Davies MM, Bath PA (2001). The maternity information concerns of Somali women in the United Kingdom. *Journal of Advanced Nursing*.

[75] Vangen S, Johansen REB, Sundby J, Træen B, Stray-Pedersen B (2004). Qualitative study of perinatal care experiences among Somali women and local health care professionals in Norway. *European Journal of Obstetrics Gynecology and Reproductive Biology*.

